# From Chronic Inflammation to Colitis-Associated Colorectal Cancer: Field Cancerization and Clonal Evolution

**DOI:** 10.3390/cancers18203247

**Published:** 2026-10-09

**Authors:** Takayuki Ogino, Yuki Sekido, Tsunekazu Mizushima, Ryota Mori, Mitsunobu Takeda, Masaaki Miyo, Atsushi Hamabe, Norikatsu Miyoshi, Mamoru Uemura, Hidetoshi Eguchi

**Affiliations:** 1 Department of Gastroenterological Surgery, The University of Osaka, Osaka 565-0871, Japan; ysekido@gesurg.med.osaka-u.ac.jp (Y.S.); rmori26@gesurg.med.osaka-u.ac.jp (R.M.); mtakeda@gesurg.med.osaka-u.ac.jp (M.T.); mmiyo@gesurg.med.osaka-u.ac.jp (M.M.); ahamabe@gesurg.med.osaka-u.ac.jp (A.H.); nmiyoshi@gesurg.med.osaka-u.ac.jp (N.M.); muemura@gesurg.med.osaka-u.ac.jp (M.U.); heguchi@gesurg.med.osaka-u.ac.jp (H.E.); 2 Department of Colorectal Surgery, Dokkyo Medical University, Tochigi 321-0293, Japan; t-mizushima123@dokkyomed.ac.jp

**Keywords:** ulcerative colitis, colorectal cancer, ulcerative colitis-associated neoplasia, dysplasia, field cancerization, clonal evolution

## Abstract

Ulcerative colitis (UC) is associated with an increased risk of colorectal cancer, particularly in patients with long-standing and extensive disease. Although advances in medical therapy and surveillance have reduced colorectal cancer incidence, prolonged preservation of chronically inflamed colonic mucosa has increased the importance of understanding how cumulative inflammation promotes carcinogenesis. UC-associated neoplasia differs from sporadic colorectal cancer in its multifocality, pathological heterogeneity, and molecular evolution. Repeated inflammation, epithelial injury, and regeneration promote the accumulation of somatic and epigenetic alterations across histologically non-dysplastic mucosa, generating a cancerized field composed of genetically heterogeneous epithelial clones. Recent sequencing studies have shown that many clones selected in chronically inflamed mucosa harbor alterations that enhance adaptation to inflammatory stress but are not necessarily premalignant, indicating that field cancerization represents a dynamic evolutionary ecosystem rather than simple expansion of cancer-prone clones. Neoplastic progression requires further clonal selection as the selective environment shifts toward autonomous growth. Colitis-associated colorectal cancer is characterized by early TP53 alterations, a relatively late and less frequent involvement of APC, branching clonal evolution, and substantial copy-number alterations, particularly during progression from low- to high-grade dysplasia. Thus, colitis-associated carcinogenesis can be understood as a process in which chronic inflammation establishes a genetically altered field, followed by dynamic clonal selection and genomic evolution. Elucidating these processes may enable identification of high-risk fields and clones before invasive cancer develops and provide a biological basis for improved surveillance, risk stratification, and chemoprevention.

## Simple Summary

Chronic inflammation in ulcerative colitis (UC) promotes the accumulation of somatic and epigenetic alterations across broad areas of the colonic mucosa, creating a cancerized field from which colitis-associated colorectal cancer (CAC) may arise. Recent genomic studies indicate that this field consists of a dynamic mosaic of epithelial clones shaped by inflammatory selection, many of which are adaptive rather than directly premalignant. Neoplastic progression follows a distinct evolutionary trajectory characterized by early TP53 alterations, branching clonal evolution, and marked copy-number changes during progression to advanced dysplasia. These findings distinguish CAC from the conventional adenoma–carcinoma sequence of sporadic colorectal cancer and provide a biological explanation for the multifocality and heterogeneity of UC-associated neoplasia. Understanding field cancerization and clonal evolution may facilitate identification of high-risk mucosa before invasive cancer develops and contribute to improved surveillance, risk stratification, and prevention strategies in patients with long-standing UC.

## 1. Introduction

Ulcerative colitis (UC) is a chronic inflammatory bowel disease characterized by a relapsing and remitting clinical course. Although its etiology remains incompletely understood, UC is thought to arise from complex interactions among genetic susceptibility, dysregulated immune responses, alterations in the gut microbiota, and environmental factors [1]. Over recent decades, substantial advances in medical therapy have markedly improved disease control and long-term outcomes, allowing an increasing proportion of patients to retain their colon for prolonged periods. Indeed, colectomy rates have declined over the past several decades, coinciding with the increasing availability and use of biologic and other advanced therapies [2].

Despite these advances, carcinogenesis associated with chronic inflammation remains one of the most important long-term complications of UC. Patients with UC remain at increased risk of colorectal cancer (CRC), particularly those with extensive and long-standing disease [3,4]. The reduction in colectomy rates represents an important achievement in contemporary UC management. However, the potential consequences of preserving chronically inflamed or inflammation-damaged colonic mucosa for decades under long-term medical therapy remain incompletely understood. In particular, whether prolonged colonic preservation affects the lifetime risk of UC-associated neoplasia (UCAN) warrants further investigation. As the population of patients living with long-standing UC continues to increase, understanding how the cumulative effects of chronic inflammation promote colorectal carcinogenesis is becoming increasingly important.

UCAN exhibits clinicopathological characteristics and molecular evolutionary patterns distinct from those of sporadic colorectal cancer (sCRC). Importantly, UC-associated carcinogenesis should not be regarded simply as a process in which prolonged inflammation eventually gives rise to a single cancer at a discrete site. Rather, repeated cycles of inflammation, epithelial injury, and regeneration may promote the accumulation of somatic molecular alterations across broad areas of the colonic mucosa, thereby generating a molecularly altered, or “cancerized,” field [5,6,7]. The concepts of field cancerization and clonal evolution therefore provide a biological framework for understanding the characteristic multifocality of UCAN and the development of synchronous (diagnosed within 6 months of each other) and metachronous (diagnosed more than 6 months apart) neoplastic lesions.

Accordingly, a central question in UC-associated carcinogenesis is how chronic inflammation establishes a cancerized field and how epithelial clones evolve within this field to ultimately give rise to colitis-associated cancer. Addressing this question requires an integrated understanding not only of the epidemiological and pathological characteristics of UCAN but also of inflammation-induced molecular alterations, field cancerization, and clonal evolution. In this narrative review, we first summarize the epidemiological and pathological characteristics of UCAN and then discuss current evidence regarding the molecular mechanisms linking chronic inflammation to field cancerization and clonal evolution. Relevant English-language articles were identified through PubMed searches focusing on UC-associated colorectal neoplasia, field cancerization, somatic and clonal evolution, and molecular mechanisms of colitis-associated carcinogenesis, supplemented by relevant references identified from selected articles. Priority was given to original genomic, molecular, pathological, and longitudinal clinical studies, together with relevant systematic reviews and consensus guidelines. Where appropriate, we distinguish findings supported by multiple independent studies from emerging observations derived from individual genomic cohorts. Finally, we consider how these emerging insights may inform cancer surveillance, risk stratification, and chemoprevention in patients with long-standing UC.

## 2. Epidemiology of UC-Associated Neoplasia

The increased risk of CRC in patients with UC has long been recognized. A landmark meta-analysis published in 2001 estimated the cumulative risk of CRC in patients with UC to be approximately 2%, 8%, and 18% at 10, 20, and 30 years after disease onset, respectively [8]. In contrast, contemporary long-term surveillance cohorts have reported cumulative CRC incidences of <1% at 10 years, approximately 3% at 20 years, and 5–7% at 30 years [9,10,11]. However, these historical and contemporary estimates should not be interpreted as directly comparable measures of temporal change, because they were derived from populations differing in referral patterns, disease severity, surveillance intensity, follow-up, and methods used to estimate cumulative cancer risk. Thus, although improvements in inflammation control and endoscopic surveillance may have contributed to a reduction in CRC risk, differences in cohort selection and methodology are also likely to account for part of the observed difference between earlier and more recent estimates. Nevertheless, the cumulative incidence of UCAN, including dysplasia, remains substantial. The cumulative incidence of dysplasia has been reported to reach 3.3%, 12.1%, and 21.8% at 10, 20, and 30 years, respectively [9]. Compared with the general population, patients with UC also remain at increased risk of both CRC and CRC-related mortality. A meta-analysis of population-based cohort studies published in 2012 reported a pooled standardized incidence ratio for CRC of 2.4 (95% confidence interval [CI], 2.1–2.7) in patients with UC [3]. More recently, a large population-based cohort study of patients with UC demonstrated increased risks of both incident CRC (hazard ratio [HR], 1.66; 95% CI, 1.57–1.76) and CRC-related death (HR, 1.59; 95% CI, 1.46–1.72) compared with matched individuals from the general population [12]. Collectively, these findings underscore the continuing importance of surveillance for UC-associated carcinogenesis despite advances in medical therapy and endoscopic surveillance. In addition to facilitating earlier detection, surveillance colonoscopy has been associated with improved survival in patients with UC-associated neoplasia; a Japanese multicenter study of surgically resected cases demonstrated significantly better overall survival among patients whose neoplasia was detected through surveillance than among those diagnosed outside a surveillance program [13].

Established risk factors for UC-associated neoplasia include longer disease duration, extensive colitis, persistent or severe inflammation, younger age at disease onset, a family history of CRC, and concomitant primary sclerosing cholangitis (PSC) [3,14]. In recent years, increasing attention has focused on cumulative inflammatory burden (CIB), which reflects long-term exposure to histological inflammation rather than disease activity at a single time point [15,16]. This concept has important biological implications for UC-associated carcinogenesis. Repeated cycles of tissue injury and epithelial regeneration may promote the accumulation of molecular alterations and the selection of epithelial clones with a survival advantage [17,18]. Indeed, molecularly altered clones have been identified in histologically non-dysplastic UC mucosa, and some of these clones extend over large areas of the colon and encompass regions in which dysplasia or carcinoma subsequently develops [6,17]. Thus, the risk of UC-associated carcinogenesis may be determined not simply by disease duration but by the magnitude and duration of inflammatory exposure throughout the disease course. Extraintestinal manifestations (EIMs) may provide additional information regarding the systemic inflammatory burden of UC, as their occurrence has been associated with more extensive disease [19]. However, EIMs have not yet been validated as a quantitative surrogate for cumulative intestinal inflammation or as an independent predictor of progression to colorectal neoplasia. At present, longitudinal histological measures such as cumulative inflammatory burden provide more direct evidence linking sustained inflammatory exposure to neoplastic risk [15,16]. Whether incorporation of EIMs into future multidimensional risk models could improve identification of patients with a high cumulative inflammatory burden warrants further investigation.

As the number of patients with long-term survival and prolonged colonic preservation continues to increase, adequate control of inflammation may be important not only for controlling UC-related symptoms but also for limiting the emergence and selection of abnormal epithelial clones that provide the substrate for neoplastic progression. At the same time, molecularly altered epithelial clones may already be present in chronically inflamed mucosa before endoscopically or histologically apparent neoplasia becomes detectable. These observations further emphasize the importance of appropriate long-term surveillance for UC-associated neoplasia.

## 3. Pathological Characteristics of UC-Associated Neoplasia

**Figure 1:**
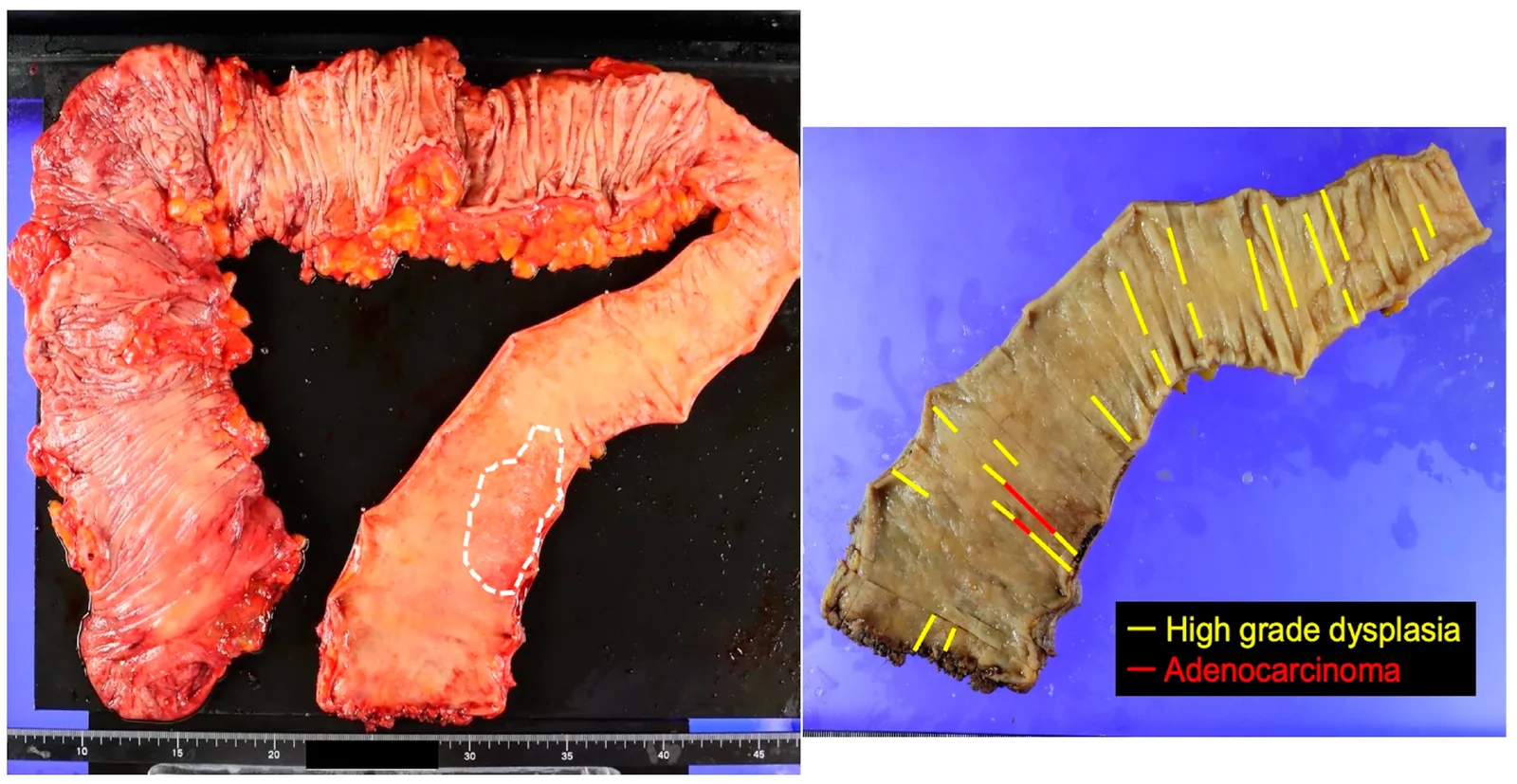
Multifocal UC-associated neoplasia. The white line indicates the gross main lesion. Pathological examination revealed multiple high-grade dysplastic lesions (yellow) and adenocarcinoma (red).

UCAN differs from sCRC in several clinicopathological respects. UCAN frequently presents as flat or non-polypoid lesions with indistinct margins and may be multifocal, making endoscopic detection and delineation challenging [20,21]. The multifocal nature of UCAN is illustrated in Figure 1, showing a colectomy specimen with adenocarcinoma and multiple spatially distinct high-grade dysplastic lesions distributed across the colonic mucosa. In particular, dysplastic changes in UCAN may involve the deeper portions of the crypts, whereas the surface epithelium may show relatively subtle abnormalities. Consequently, diagnostic approaches relying predominantly on surface architecture, which are highly useful for sporadic adenomas and carcinomas, may have limitations in UCAN. In this setting, chronically inflamed colonic mucosa should be considered a field at risk for neoplastic transformation [6,22].

Histologically, UC-associated dysplasia exhibits considerable morphological heterogeneity. In addition to conventional intestinal (adenoma-like) dysplasia, several non-conventional subtypes have been described, including hypermucinous dysplasia, goblet cell-deficient dysplasia, crypt cell dysplasia, dysplasia with increased Paneth cell differentiation, and several forms of serrated dysplasia [23,24]. More recently, an international consensus classification categorized inflammatory bowel disease (IBD)-associated dysplasia into three broad categories—intestinal, gastric, and mixed intestinal–gastric dysplasia—with further subdivision according to specific morphological features [25]. Because of this marked histological heterogeneity and the background of chronic inflammation and regeneration, distinguishing UC-associated dysplasia from regenerative or reactive atypia can occasionally be challenging [21,25].

p53 immunohistochemistry plays an important ancillary role in the diagnosis of UC-associated dysplasia. Whereas TP53 alterations generally occur relatively late in the conventional adenoma–carcinoma sequence of sporadic CRC, TP53 abnormalities can arise early in UC-associated tumorigenesis [18,26]. Aberrant p53 expression may therefore be detectable in dysplastic lesions before the development of invasive carcinoma and can assist in distinguishing neoplastic lesions from regenerative atypia, although p53 immunohistochemistry should always be interpreted in conjunction with histomorphology [27,28,29]. Importantly, TP53 abnormalities may also be detected in chronically inflamed mucosa without histologically recognizable dysplasia [6,18,30]. This observation indicates that molecularly altered epithelial clones can arise before the emergence of morphologically evident neoplasia. Indeed, clonal TP53 mutations have been demonstrated in non-dysplastic crypts surrounding UC-associated neoplasms, providing direct evidence for local or segmental field cancerization [6]. Thus, TP53 abnormalities may represent not only molecular alterations associated with dysplasia but also manifestations of the cancerized field from which UC-associated neoplasia develops.

The distribution of proliferative and cell-cycle abnormalities within colonic crypts provides additional diagnostic information. UC-associated dysplasia frequently exhibits a “bottom-up” pattern of proliferation, in which the proliferative zone is preferentially located in the deeper portions of the crypts, whereas sporadic adenomas characteristically show a more superficial or “top-down” pattern [31]. Ki-67 immunohistochemistry can provide useful ancillary information in this setting. In UC-associated dysplasia, extension of Ki-67-positive cells beyond the basal third of the crypt supports the presence of dysplasia, whereas extension of Ki-67 expression and strong p53 staining into the upper portions of the crypts is associated with higher-grade dysplasia [29]. Aberrant p53 immunostaining may manifest as either diffuse strong nuclear overexpression or complete loss of expression. However, because p53 and Ki-67 staining patterns may overlap with those observed in reactive or regenerative epithelium, both should be regarded as ancillary rather than stand-alone diagnostic tests and should always be interpreted in conjunction with histomorphology and the broader clinicopathological context [20,29].

Taken together, the flat and poorly demarcated morphology, multifocality, histological heterogeneity, and early occurrence of TP53 abnormalities characteristic of UCAN support a model in which carcinogenesis does not simply arise as an isolated event from a single transformed epithelial cell. Rather, neoplastic evolution occurs within a cancerized field generated in chronically inflamed colonic mucosa [6,18,22]. A key question, therefore, is how this field is established and how distinct epithelial clones are subsequently selected, expanded, and evolutionarily shaped within it, ultimately giving rise to dysplasia and invasive carcinoma.

## 4. Field Cancerization

**Figure 2:**
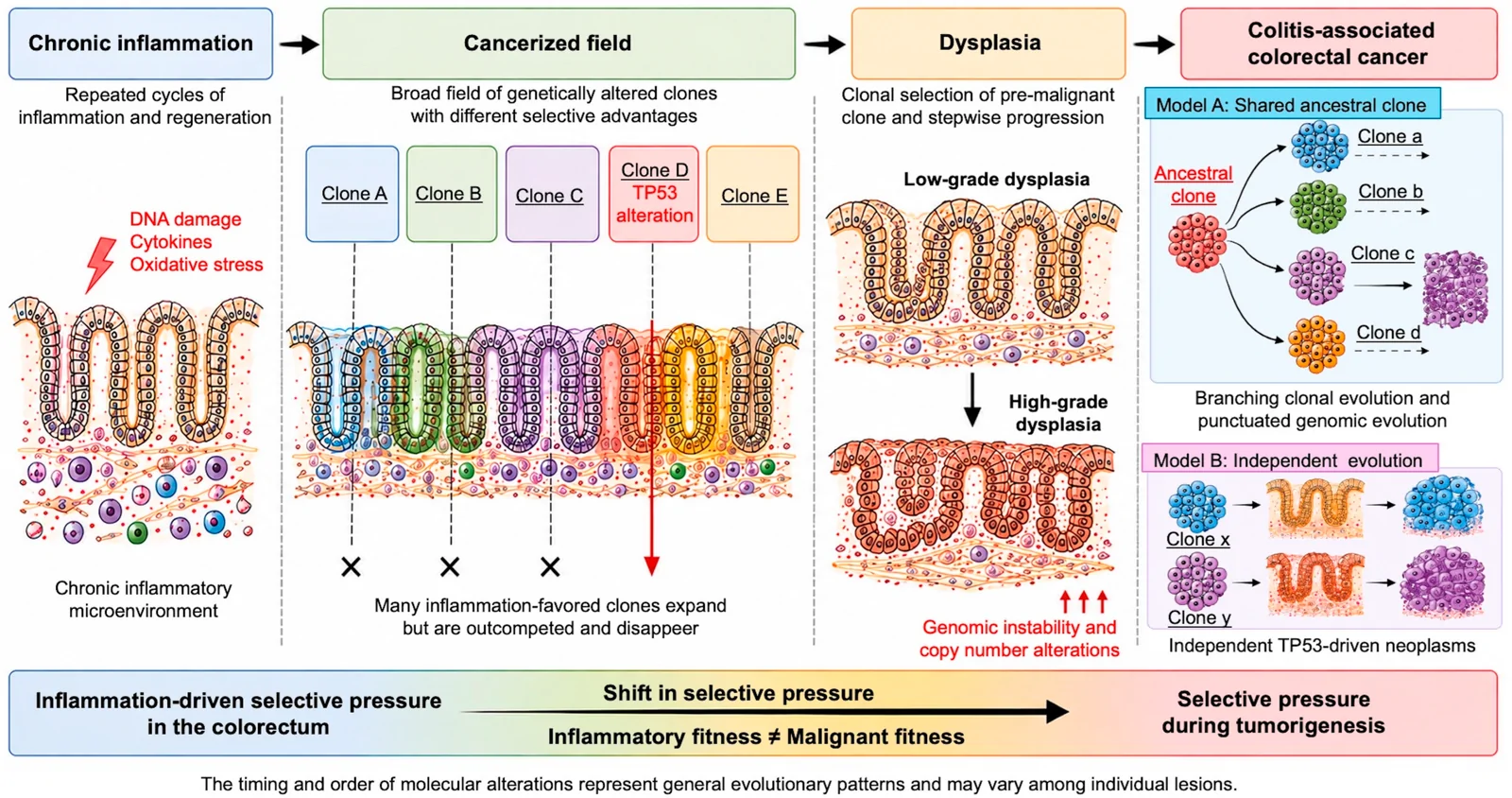
Field cancerization and clonal evolution in ulcerative colitis-associated colorectal cancer.

Colitis-associated colorectal cancer (CAC) is thought to arise through the molecular evolution of colonic epithelial cells under conditions of chronic inflammation. A key concept underlying this process is field cancerization, in which genetically altered epithelial clones expand within histologically non-dysplastic mucosa, creating a field predisposed to neoplastic transformation [6,32] (Figure 2). In UC, both the duration and severity of inflammation are associated with an increased risk of colorectal neoplasia, supporting the concept that chronic inflammation promotes somatic evolution of the colonic epithelium [33,34]. Longitudinal studies have further shown that aneuploid cell populations can persist and expand spatially before histologically apparent dysplasia develops [35].

Single-crypt sequencing has demonstrated that non-neoplastic IBD-affected epithelium carries a substantially higher somatic mutation burden than healthy colonic epithelium. Importantly, this increase appears to result largely from the acceleration of mutational processes already operating in normal colonic epithelium, rather than from a distinct IBD-specific mutational process [36]. Thus, chronic inflammation may accelerate epithelial turnover and somatic evolution, resulting in extensive clonal remodeling of histologically normal-appearing mucosa. This genetically heterogeneous mosaic provides the biological basis for field cancerization and may contribute to the development of synchronous and metachronous neoplasia in UC [6,36]. However, because the single-crypt sequencing findings were derived from an IBD population that included both UC and Crohn’s disease, their applicability specifically to UC should be interpreted with appropriate caution.

Importantly, however, clonal expansion in inflamed mucosa does not necessarily represent premalignant evolution. Recent sequencing studies have identified recurrent mutations in NFKBIZ, PIGR, ZC3H12A, TRAF3IP2, and other genes involved in IL-17/NF-κB signaling in non-dysplastic UC epithelium [37,38]. Many of these alterations attenuate inflammatory signaling and confer a selective advantage under chronic inflammatory stress. Remarkably, some mutations that are strongly selected in inflamed mucosa, particularly NFKBIZ alterations, are uncommon in established CAC [37]. These findings indicate that clones adapted to the inflammatory environment are not necessarily those that ultimately undergo malignant transformation. Thus, the UC-associated cancerized field should be regarded not simply as the widespread expansion of premalignant clones but as a dynamic mosaic of genetically distinct clones subjected to competing selective pressures.

Clinically, this field effect provides a biological rationale for considering dysplasia in UC as a marker of risk extending beyond the individual lesion. Nevertheless, current management is determined by the grade, visibility, and focality of dysplasia rather than by the concept of field cancerization alone. Invisible high-grade dysplasia, multifocal dysplasia, and endoscopically unresectable lesions generally favor colectomy, whereas completely resectable visible lesions may be managed endoscopically with close surveillance in selected patients [39]. A central unresolved question is how selection pressures within the inflamed field change over time and ultimately favor the emergence of clones capable of progressing to CAC.

## 5. Molecular Mechanisms of Colitis-Associated Carcinogenesis

CAC develops through interactions between chronic inflammation and genetic and epigenetic alterations in colonic epithelial cells. Persistent inflammation exposes the epithelium to reactive oxygen and nitrogen species generated by activated immune cells, resulting in DNA damage and genomic instability [40,41]. In parallel, inflammation-associated epigenetic alterations, particularly aberrant DNA methylation, may alter the expression of genes involved in tumor suppression and epithelial homeostasis [42]. Pro-inflammatory cytokines, including TNF-α, IL-6, IL-17, and IL-23, further activate tumor-promoting pathways such as NF-κB and JAK/STAT3, thereby enhancing epithelial survival and proliferation and linking chronic inflammation to neoplastic progression [41].

The gut microbiota and mucosal immune system may also contribute to shaping the inflammatory environment in which field cancerization develops. Group 3 innate lymphoid cells (ILC3s) are abundant at intestinal mucosal surfaces and participate in epithelial homeostasis and host–microbiota interactions through cytokines including IL-17 and IL-22. Single-cell analyses have demonstrated that ILC3s are markedly dysregulated in intestinal inflammation and CRC, with reduced frequencies, altered cellular plasticity, and disrupted interactions with T cells. In particular, MHC-II-dependent interactions between ILC3s and T cells contribute to the maintenance of a microbiota that supports type 1 immunity, whereas disruption of this interaction promotes intestinal inflammation and invasive CRC in experimental models [43]. In human CRC, we previously demonstrated that NKp44+ ILC3s expressing genes involved in tertiary lymphoid structure formation were enriched in normal mucosa and early-stage tumors but decreased during tumor progression in association with reduced tertiary lymphoid structure density [44]. These findings suggest that dysregulation of the microbiota–ILC3–adaptive immune axis may modify the inflammatory and immune-selective environment of the colonic mucosa and thereby contribute to the establishment and evolution of the UC-associated cancerized field.

CAC shares the major forms of genomic instability observed in sCRC, including chromosomal instability (CIN), microsatellite instability (MSI), and aberrant DNA methylation, although their timing and relative contributions differ [45,46]. Chromosomal alterations may occur remarkably early in UC-associated carcinogenesis: clonal chromosomal abnormalities have been reported in 85% of cancers, 86% of dysplastic lesions, and even 36% of histologically non-dysplastic mucosal samples from colectomy specimens containing UC-associated cancer [45]. Microsatellite instability-high (MSI-H) occurs in a smaller subset of CAC and may also be detected at the dysplastic stage [45]. These observations indicate that molecular abnormalities can precede morphologically recognizable dysplasia and are consistent with the field cancerization model described above.

Although CAC and sCRC share several driver genes, including TP53, KRAS, APC, PIK3CA, and SMAD4, the frequencies and temporal sequence of these alterations differ substantially [46,47,48]. In the conventional sporadic adenoma–carcinoma sequence, APC alteration is typically an early event, followed by additional alterations such as KRAS activation and TP53 loss. In contrast, TP53 alterations frequently occur early in CAC and may be detected before histologically recognizable dysplasia, whereas APC alterations are less frequent and, when present, tend to occur later [46,47,48]. These patterns should be regarded as general features of CAC evolution rather than a universal molecular sequence, because the timing, order, and combination of individual driver alterations may vary among lesions. Large genomic studies have confirmed TP53 as the most frequently altered gene in CAC, whereas APC alterations occur in only approximately 20% of tumors [47,48]. Wnt signaling nevertheless remains dysregulated in CAC, suggesting mechanisms distinct from the classical APC-driven pathway of sCRC [48,49]. These differences in the timing and frequency of driver alterations indicate that CAC follows an evolutionary trajectory distinct from the conventional adenoma–carcinoma sequence of sCRC.

Transcriptomic studies further support the molecular distinction between IBD-associated CRC and sCRC. In an integrated genomic, epigenomic, and transcriptomic analysis, IBD-associated CRCs, including tumors arising in both UC and Crohn’s disease, lacked the canonical Wnt-driven CMS2 phenotype and were instead enriched for a mesenchymal, stroma-rich phenotype resembling CMS4, characterized by epithelial–mesenchymal transition and stromal activation [49]. This shift may reflect the chronic inflammatory and fibrotic microenvironment characteristic of IBD. However, because the study population included both UC and Crohn’s disease rather than being restricted to UC, the direct applicability of these findings to UC-associated carcinogenesis requires further validation.

## 6. Clonal Evolution

Field cancerization creates a genetically heterogeneous mosaic of epithelial clones within histologically non-dysplastic UC mucosa. However, only a minority of these clones ultimately progress to CAC. Carcinogenesis therefore requires further clonal selection as the evolutionary landscape changes during progression from chronically inflamed mucosa to dysplasia and carcinoma.

As discussed above, TP53 alteration is a characteristic early event in CAC evolution. Multiregion and genomic studies have demonstrated that TP53 alterations are already present in dysplasia and may be shared across large regions of an individual neoplasm, consistent with their acquisition early in the evolutionary history of the tumor [50,51]. This pattern is compatible with spatial expansion and subsequent diversification from a shared ancestral clone. However, distinct dysplastic or cancerous lesions arising within the same inflamed colon may harbor independent TP53 alterations, supporting an alternative pattern in which multiple neoplastic clones emerge independently within the cancerized field [51]. Thus, UC-associated neoplasia may arise through both shared clonal expansion and independent, convergent evolutionary trajectories rather than through a single universal sequence of molecular events. Additional alterations involving KRAS, SMAD4, copy-number changes, and other signaling pathways generate genetically distinct subclones, supporting a branching rather than a fixed linear model of CAC progression.

A particularly important feature of CAC evolution is the acquisition of chromosomal copy-number alterations (CNAs). In a multiregion genomic analysis, Baker et al. demonstrated that CNAs can already be detected in non-dysplastic mucosa and low-grade dysplasia (LGD), but that progression from LGD to high-grade dysplasia (HGD) is frequently accompanied by a dramatic, punctuated increase in CNA burden. In contrast, relatively little additional increase is observed between HGD and invasive carcinoma [50]. Although these findings provide important evidence for punctuated genomic evolution during CAC progression, they were derived from a relatively limited genomic cohort and require validation in independent longitudinal cohorts. These findings suggest that much of the genomic architecture of CAC is established before invasion, during the transition to advanced dysplasia. This pattern is consistent with punctuated genomic evolution, in which extensive chromosomal restructuring occurs during a relatively restricted phase of tumor evolution rather than through the gradual accumulation of genomic abnormalities [52].

As disease progresses, the selective landscape shifts from adaptation to chronic inflammation toward autonomous proliferation and malignant growth, thereby favoring a distinct subset of clones capable of neoplastic progression. CAC evolution can therefore be viewed as a branching and partly punctuated process in which early neoplastic clones emerge within a genetically heterogeneous field and subsequently undergo further selection and genomic diversification, particularly during progression to advanced dysplasia, as summarized in Figure 2.

## 7. Conclusions

Chronic inflammation in UC promotes the formation of a cancerized field composed of multiple genetically and epigenetically altered epithelial clones. Importantly, clonal expansion within this field does not necessarily signify premalignant evolution. Rather, changing selective pressures continually reshape clonal composition, and only a subset of clones ultimately acquires the capacity for neoplastic progression. Early TP53 alterations, branching clonal evolution, and marked copy-number changes during progression to advanced dysplasia further characterize the evolutionary trajectory toward CAC. Thus, CAC is best understood as the consequence of field cancerization followed by dynamic clonal selection and genomic evolution within chronically inflamed mucosa. Understanding this process may provide a framework for identifying high-risk fields and clones before the development of invasive cancer and for improving surveillance and prevention strategies in patients with UC. At present, however, clinical decisions regarding surveillance and colectomy remain based on established endoscopic and histopathological findings, as no molecular assay has yet been prospectively validated to guide these decisions. Molecular characterization of high-risk mucosal fields remains a promising strategy for future risk stratification but requires prospective validation before incorporation into routine clinical practice.

## Data Availability

No new data were created or analyzed in this study. Data sharing is not applicable to this article.

## Abbreviations

The following abbreviations are used in this manuscript:

CAC	Colitis-associated colorectal cancer
CIB	Cumulative inflammatory burden
CI	Confidence interval
CIN	Chromosomal instability
CMS	Consensus molecular subtype
CNA	Copy-number alteration
CRC	Colorectal cancer
HGD	High-grade dysplasia
HR	Hazard ratio
IBD	Inflammatory bowel disease
LGD	Low-grade dysplasia
MSI	Microsatellite instability
MSI-H	Microsatellite instability-high
PSC	Primary sclerosing cholangitis
sCRC	Sporadic colorectal cancer
UC	Ulcerative colitis
UCAN	Ulcerative colitis-associated neoplasia
